# Pandemic potential of highly pathogenic avian influenza clade 2.3.4.4 A(H5) viruses

**DOI:** 10.1002/rmv.2099

**Published:** 2020-03-05

**Authors:** Reina Yamaji, Magdi D. Saad, Charles T. Davis, David E. Swayne, Dayan Wang, Frank Y.K. Wong, John W. McCauley, J.S. Malik Peiris, Richard J. Webby, Ron A.M. Fouchier, Yoshihiro Kawaoka, Wenqing Zhang

**Affiliations:** ^1^ Global Influenza Programme, Infectious Hazards Management, WHO Emergency Programme, WHO Geneva Switzerland; ^2^ Influenza Division National Center for Immunization and Respiratory Diseases, Centers for Disease Control and Prevention Atlanta Georgia USA; ^3^ Department of Agriculture OIE Collaborating Centre for Research on Emerging Avian Diseases, U.S. National Poultry Research Center, Agricultural Research Service Athens Georgia USA; ^4^ National Institute for Viral Disease Control and Prevention China; ^5^ CSIRO Australian Animal Health Laboratory Geelong Australia; ^6^ WHO Collaborating Centre for Reference and Research on Influenza, Crick Worldwide Influenza Centre, The Francis Crick Institute London UK; ^7^ School of Public Health, Li Ka Shing Faculty of Medicine, The University of Hong Kong, Hong Kong Special Administrative Region China; ^8^ Department of Infectious Diseases St. Jude Children's Research Hospital Memphis Tennessee USA; ^9^ Department of Viroscience Erasmus MC Rotterdam The Netherlands; ^10^ Division of Virology, Department of Microbiology and Immunology Institute of Medical Science, University of Tokyo Tokyo Japan; ^11^ Department of Pathobiological Sciences School of Veterinary Medicine, University of Wisconsin‐Madison Madison Wisconsin USA; ^12^ Department of Special Pathogens, International Research Center for Infectious Diseases Institute of Medical Science, University of Tokyo Tokyo Japan

**Keywords:** avian influenza, zoonosis, zoonotic influenza

## Abstract

The panzootic caused by A/goose/Guangdong/1/96‐lineage highly pathogenic avian influenza (HPAI) A(H5) viruses has occurred in multiple waves since 1996. From 2013 onwards, clade 2.3.4.4 viruses of subtypes A(H5N2), A(H5N6), and A(H5N8) emerged to cause panzootic waves of unprecedented magnitude among avian species accompanied by severe losses to the poultry industry around the world. Clade 2.3.4.4 A(H5) viruses have expanded in distinct geographical and evolutionary pathways likely via long distance migratory bird dispersal onto several continents and by poultry trade among neighboring countries. Coupled with regional circulation, the viruses have evolved further by reassorting with local viruses. As of February 2019, there have been 23 cases of humans infected with clade 2.3.4.4 H5N6 viruses, 16 (70%) of which had fatal outcomes. To date, no HPAI A(H5) virus has caused sustainable human‐to‐human transmission. However, due to the lack of population immunity in humans and ongoing evolution of the virus, there is a continuing risk that clade 2.3.4.4 A(H5) viruses could cause an influenza pandemic if the ability to transmit efficiently among humans was gained. Therefore, multisectoral collaborations among the animal, environmental, and public health sectors are essential to conduct risk assessments and develop countermeasures to prevent disease and to control spread. In this article, we describe an assessment of the likelihood of clade 2.3.4.4 A(H5) viruses gaining human‐to‐human transmissibility and impact on human health should such human‐to‐human transmission occur. This structured analysis assessed properties of the virus, attributes of the human population, and ecology and epidemiology of these viruses in animal hosts.

**Abbreviations**
HPAI: Highly Pathogenic Avian Influenza
LPAI: Low Pathogenicity Avian Influenza
WHO: World Health Organization
GIP: Global Influenza Programme
TIPRA: Tool for Influenza Pandemic Risk Assessment
IRAT: Influenza Risk Assessment Tool
CDC: United States Centers for Disease Control and Prevention
GISRS: WHO Global Influenza Surveillance and Response System
OIE: World Organization for Animal Health
FAO: Food and Agriculture Organization
HA: Hemagglutinin
ROK: Republic of Korea
CVV: WHO Candidate Vaccine Virus
USDA: United States Department of Agriculture
ARDS: Acute Respiratory Distress Syndrome
MOF: Multiple Organ Failure
Sia‐α2,6Gal: Sialic Acid Linked to Galactose by an α2,6 linkage
3′SLeX: Sialyl Lewis X
GOF: Gain‐Of‐Function
GISAID: Global Initiative on Sharing All Influenza Data
GSD: Genetic Sequence Data

## 1 INTRODUCTION

Influenza A viruses infect a wide spectrum of animal species precluding global eradication. Genetically diverse viruses circulate among wild aquatic birds, which are considered to be their natural reservoir and experience no or only mild signs of disease when infected. In birds, the viruses typically replicate in the intestinal and respiratory tracts and are shed in the environment where other hosts become infected. Viruses from the aquatic wild bird reservoir may infect other avian species including terrestrial poultry, such as chickens and quail, and domesticated waterfowl, such as ducks and geese. Following circulation in these densely populated host species, avian influenza viruses may then transmit to mammalian hosts, including humans, pigs, horses, dogs, and marine mammals.^1^

Globalization and industrialization over the past decades have contributed to the emergence of novel influenza viruses that threaten animal and human health. Once they emerge and become transmissible between humans, influenza viruses can rapidly spread worldwide. Current vaccines which take 6 months to distribute from strain selection in the current influenza manufacturing cycle are unlikely to be available to contain the first wave of human infections of a pandemic. Therefore, it is strategically important to risk‐assess and prioritize animal influenza viruses with pandemic potential to initiate possible responses, including preparatory development of vaccines, and antiviral drug efficacy testing. The World Health Organization (WHO) Global Influenza Programme (GIP) developed a Tool for Influenza Pandemic Risk Assessment (TIPRA)^2^ based on the Influenza Risk Assessment Tool (IRAT)^3^ developed by the WHO Collaborating Centre at the United States Centers for Disease Control and Prevention (CDC) and in consultation with experts in the WHO Global Influenza Surveillance and Response System (GISRS) and other institutions and academia. Since TIPRA was launched in 2016, it has provided a framework for influenza A virus risk assessment through a standardized approach for evaluating the likelihood of pandemic emergence and associated impact of a novel virus. In this risk assessment process, the WHO, the World Organization for Animal Health (OIE), and the Food and Agriculture Organization (FAO) tripartite collaboration brings together multiple stakeholders worldwide, including public and animal health practitioners and influenza researchers from different sectors within the “One Health” concept, and strengthens interdisciplinary global collaboration.^4^

Because the emergence of the highly pathogenic avian influenza (HPAI) A(H5) viruses of the A/goose/Guangdong/1/96 (gs/GD) hemagglutinin (HA) lineage, there have been 883 officially reported human infections by viruses of this lineage: 860 by A(H5N1) and 23 by A(H5N6) viruses.^5^ The dominant HA clades of H5 viruses vary temporally and spatially, with some achieving a wide geographical spread. Infections among humans and other mammals,^6^, ^7^, ^8^ however, have been restricted to the initial index cases or a small number of close contacts. Because HPAI A(H5) viruses bearing an HA of clade 2.3.4 were identified in China in 2008, they have evolved into further subgroups including clade 2.3.4.4^9^ and have acquired various neuraminidase subtypes, including N1, N2, N5, N6, and N8, by reassortment with other avian influenza viruses enzootic in different regions. In addition, the geographic spread of clade 2.3.4.4 A(H5) viruses has been unprecedented, resulting in regional epizootics in poultry, increasing the opportunities for avian‐to‐human transmission. Although human‐to‐human transmission of clade 2.3.4.4 A(H5) viruses has not been observed to date, the pandemic potential of these viruses remains unpredictable. Given the lack of population immunity to A(H5) subtype viruses, the ongoing evolution of clade 2.3.4.4 A(H5) viruses, and sporadic human infections, the pandemic potential of these viruses cannot be ignored. In this review, we focus on the three clade 2.3.4.4 subtypes, A(H5N2), A(H5N6), and A(H5N8), that have the greatest frequency of global detections, and describe their biological features and the use of TIPRA in risk asessment.^2^

## 2 GLOBAL SPREAD OF HPAI CLADE 2.3.4.4 A(H5) VIRUSES

**Figure 1:**
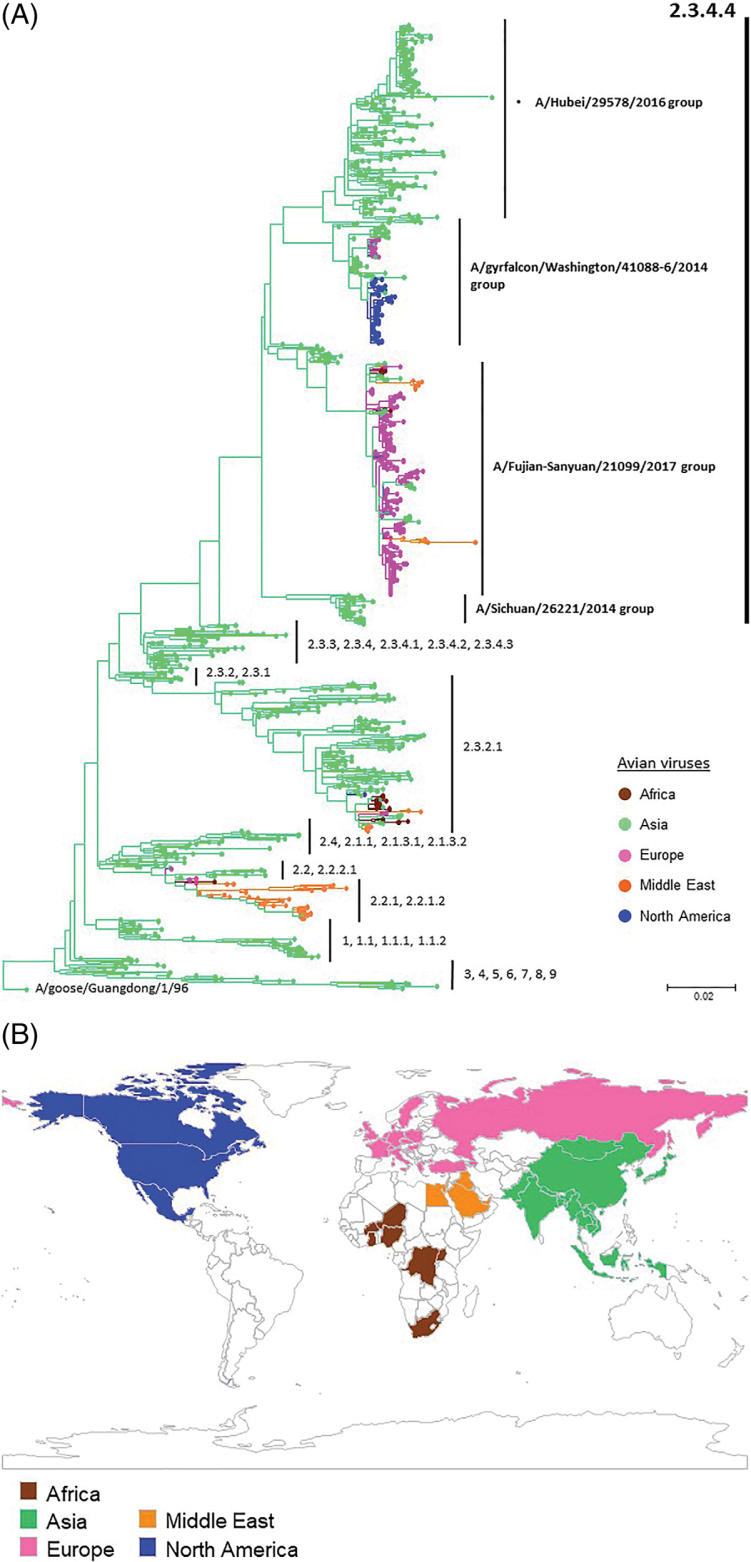
(a) Geographical regions in the world that confirmed to have isolated clade 2.3.4.4 A(H5) viruses from animals; mammals and avian species. Geographical regions colored in brown, Africa; green, Asia; pink, Europe; orange, Middle East; and blue, North and South America. (b) Phylogenetic relationships of HA genes of A(H5) highly pathogenic avian influenza viruses. Of 3685 HPAI A(H5) viruses isolated from animals including mammals and avian species available in Global Initiative on Sharing All Influenza Data (GISAID) and GenBank between 2013 and 2018, arbitrarily chosen 1134 strains were analyzed. The open reading frame of HA genes A(H5) virus was used for phylogenetic analysis. Multiple sequence alignment of A(H5) viruses was performed together with alignment of genetic sequence data (GSD) downloaded from GISAID using BioEdit 7.2. A maximum‐likelihood tree using the 1134 A(H5) HA genes and 242 representative A(H5) HA genes^135^ rooted to A/goose/Guangdong/1/96 was constructed for MEGA 7 with 1000 replicate

The clade 2.3.4.4 A(H5N8) viruses were first reported in migratory ducks and curlews in Shanghai, China in 2013 by retrospective surveillance,^10^ followed by outbreaks in the Republic of Korea (ROK) in January of 2014.^11^, ^12^, ^13^, ^14^ During the outbreaks in ROK, two distinct genetic groups were identified: a group represented by A/broiler duck/Korea/Buan2/2014 and the WHO candidate vaccine virus (CVV) recommended by WHO,^15^ A/gyrfalcon/Washington/41088‐6/2014 (referred to as “group A” by Lee et al^16^), and another group represented by A/breeder duck/Korea/Gochang1/2014^14^ and the CVV, A/Fujian‐Sanyuan/21099/2017 (referred to as “group B” by Lee et al^16^) (Figure 1a). A group of viruses represented by A/gyrfalcon/Washington/41088‐6/2014 (hereby A/gyrfalcon/Washington/41088‐6/2014 group) likely spread eastwards, to North America via Beringia by long‐distance migratory birds^17^, ^18^, ^19^, ^20^, ^21^, ^22^ (Figure 1b). In November 2014, these viruses reassorted with avian influenza viruses from North American wild birds generating an A(H5N2) virus that was the cause of an outbreak in poultry farms in British Columbia.^23^ From March through mid‐June of 2015, HPAI A(H5N2) viruses caused widespread outbreaks in commercial poultry flocks mainly in the Pacific, Western, and North Central regions of the United States.^24^ The spread of the virus in the United States was accompanied by multiple reassortment events between HPAI A(H5) viruses and low pathogenicity avian influenza (LPAI) viruses from wild and domestic birds.^25^ The United States Department of Agriculture (USDA) documented that during the outbreaks 50.4 million birds died or were culled in the 15 affected states.^17^, ^20^, ^26^, ^27^, ^28^ After the initial wave of outbreaks in North America, detections of the HPAI A(H5) virus declined; it has not been detected in poultry since June 16, 2015 or in wild birds since December 16, 2016 in North America^25^, ^29^, ^30^ (Supplementary Table 1). In parallel with the spread of clade 2.3.4.4 A(H5N8) viruses to North America, related A/gyrfalcon/Washington/41088‐6/2014‐group viruses had also moved into Europe and were widespread by the end of 2014.^31^, ^32^, ^33^, ^34^, ^35^ Nevertheless, it is noteworthy that sporadic outbreaks of A/gyrfalcon/Washington/41088‐6/2014‐group A(H5N2) and A(H5N8) viruses continue to be detected among poultry with the latest outbreak caused by A(H5N2) viruses in a chicken farm in April 2019.^36^

Although the A/gyrfalcon/Washington/41088‐6/2014‐group viruses were disseminated from Asia to other continents, a group of viruses represented by A/Fujian‐Sanyuan/21099/2017 (hereby A/Fujian‐Sanyuan/21099/2017 group) did not initially appear to spread outside Asia.^37^ However, this changed in mid‐2016 when these A(H5N8) viruses were detected in wild birds at Tyva Republic near Uvs‐Nuur Lake in Russian Federation^38^, ^39^, ^40^ and Qinghai Lake in China.^41^ This group subsequently spread, presumably by wild birds, to many other countries^19^, ^22^, ^42^ in Africa,^43^, ^44^, ^45^ Asia,^46^, ^47^, ^48^, ^49^, ^50^ Europe,^51^, ^52^, ^53^, ^54^, ^55^, ^56^, ^57^ and the Middle East^58^, ^59^, ^60^, ^61^, ^62^ (Figure 1b). As was seen with the earlier spread of A(H5N8) viruses in North America, multiple reassortment events with local wild bird viruses occurred generating additional NA subtypes.^63^, ^64^ According to the OIE, between mid‐2016 and October 2018, 51 countries in Africa, Asia, and Europe reported clade 2.3.4.4 A(H5N8) viruses in either poultry or wild birds.^65^ From 2017‐2018, A(H5N6) viruses with HA gene of the A/Fujian‐Sanyuan/21099/2017 group were isolated from birds in the Netherlands,^66^ the United Kingdom,^67^ Germany, Greece, Republic of Georgia, and Denmark and Eastern Asian countries^48^, ^68^, ^69^ (Supplementary Table 1).

In contrast to the A/gyrfalcon/Washington/41088‐6/2014‐group and A/Fujian‐Sanyuan/21099/2017‐group viruses, which were characterized by global spread, other genetic groups of clade 2.3.4.4 A(H5) viruses have to date remained more limited in geographic range. One group represented by the WHO CVVs, A/Hubei/29578/2016, A/chicken/Vietnam/NCVD‐15A59/2015, and A/duck/Hyogo/1/2016 (referred to as “group C” by Lee et al^16^ and hereby A/Hubei/29578/2016 group), is comprised mainly A(H5N6) viruses that have been maintained among avian species since 2013 in China,^70^, ^71^ Japan,^72^, ^73^, ^74^ Lao People's Democratic Republic,^75^ ROK,^76^ and Vietnam^77^ (Figure 1a and b). The viral ancestors to several groups of clade 2.3.4.4 A(H5) viruses described herein are represented by the WHO CVV, A/Sichuan/26221/2014 (referred to as “group D” by Lee et al^16^ and hereby A/Sichuan/26221/2014 group). This group of primarily A(H5N6) viruses were identified in China as early as 2010,^78^, ^79^, ^80^, ^81^ but have not been detected since 2015.

## 3 INFECTIONS IN MAMMALS AND IN ANIMAL MODELS

Clade 2.3.4.4 A(H5N2), A(H5N6), and A(H5N8) with genetic groups have been identified from both poultry and wild birds.^82^, ^83^ Detections of A(H5N6) viruses in cats and pigs have been reported^6^, ^7^, ^8^; at least two of these had epidemiological links with infected poultry or an infected human.^6^, ^7^ In one instance, an A(H5N6) virus was detected from a dead cat found in proximity to the residence of a patient infected with an A/Sichuan/26221/2014‐group A(H5N6) virus in Sichuan province, China.^6^ An A(H5N6) virus was also isolated from a nasal swab taken from a pig in Guangdong province, China, in 2014 and was found to be closely related to A(H5N6) viruses isolated from ducks in the area at the same time.^7^ In addition, an A(H5N6) virus isolated from a cat carcass in Zhejiang province, China, in 2016 was found to share three gene segments, HA, NA, and PA, with A/Hubei/29578/2016‐group A(H5N6) viruses co‐circulating in eastern and southern China in 2013‐2016; the other five genes were closely related to A(H9N2) and A(H7N9) viruses.^8^ In contrast to the A(H5N6) viruses, natural infections of mammalian species by clade 2.3.4.4 A(H5N2) and A(H5N8) viruses have not yet been detected.

Several studies have documented the enhanced virulence of some clade 2.3.4.4 A(H5) viruses in experimentally infected mammals.^84^, ^85^, ^86^, ^87^ An A(H5N6) virus isolated from a patient who had underlying medical conditions and recovered from severe pneumonia, A/Guangzhou/39715/2014, with the E627K substitution in the PB2 protein, produced severe pneumonia in ferrets inoculated intra‐tracheally with 10^6^ TCID_50_ of the virus.^86^ However, in several reports, clade 2.3.4.4 A(H5) viruses showed mild disease with no mortality in experimentally inoculated ferrets.^88^, ^89^, ^90^, ^91^ Two studies showed that the pathogenicity of clade 2.3.4.4 A(H5N8) viruses in ferrets were milder than control HPAI A(H5N1) viruses.^91^, ^92^ Although the ferret model has an advantage of displaying similar clinical manifestation of influenza virus infection to those of humans, there are limited experimental data available due to disadvantages such as high cost and laborious handling. Although susceptibility to influenza virus infection of mice varies with their genetic background and its clinical manifestations are dissimilar to those of typical influenza virus infection of humans, mice are an established model to assess the pathogenicity of influenza virus. An A(H5N8) A/Fujian‐Sanyuan/21099/2017‐group virus caused 100% mortality in mice when intra‐nasally inoculated at a dose of 10^6.0^ EID_50_, despite the lack of the well characterized mammalian pathogenicity markers PB2 627K and 701N.^87^ Although some clade 2.3.4.4 A(H5) viruses can cause severe disease in experimentally infected mammals, several studies showed considerable variation in pathogenicity.^93^, ^94^, ^95^ One A/Hubei/29578/2016‐group A(H5N6) virus showed enhanced virulence in mice with a mortality rate of 80%, whereas mice infected with three other viruses of the same subtype and group survived the 14‐day observation period.^94^ Similarly, Zhao et al. showed that three A(H5N6) viruses exhibited different pathogenicity in mice following intra‐nasal inoculation with 10^6^ EID_50_ of virus; two viruses caused 60% mortality, whereas the other was not lethal.^95^ Dogs intra‐nasally inoculated with 10^6^ EID_50_ of an A(H5N6) virus shed virus for 7 days with no mortality, similar to what was observed with a control HPAI A(H5N1) virus.^96^ However, the extent to which common laboratory mammalian models can predict the pathogenicity of influenza viruses in humans or even the replication in human cells remains unclear. For example, Grund et al. demonstrated that a A/Fujian‐Sanyuan/21099/2017‐group A(H5N8) virus was highly pathogenic for mice without prior adaptation; however, the same virus replicated poorly in human lung explants.^97^

## 4 HUMAN INFECTIONS WITH CLADE 2.3.4.4 A(H5N6) VIRUS

The first human infection caused by a clade 2.3.4.4 A(H5N6) virus was reported by China in April 2014.^98^, ^99^, ^100^, ^101^, ^102^ As of February 2019, a total of 23 human infections with A(H5N6) viruses were reported to WHO, 16 (70%) of which had fatal outcomes. Eighteen of the total human infections (78%) were reported in 2014‐2016, one in 2017, and four in 2018. Most infections occurred in the southern China provinces. According to the self‐reported exposure history of people infected with HPAI A(H5N6) virus, 19 of 23 had exposure to poultry, which therefore suggested that contact with poultry or contaminated poultry market environments was the source of infection.^100^, ^103^, ^104^, ^105^, ^106^, ^107^, ^108^ The hospitalized patients initially showed influenza‐like symptoms including fever, sore throat, headache, chills, cough, and myalgia, then developed into shortness of breath due to severe pneumonia and progressed to acute respiratory distress syndrome (ARDS) and multiple organ failure (MOF) in the deceased patients.^99^, ^100^, ^101^, ^103^, ^104^, ^105^, ^106^, ^107^, ^108^, ^109^, ^110^ Bi et al. indicated that A(H5N6) patients were observed to have significantly higher levels of 11 cytokines and 5 chemokines among the 48 markers tested, compared to individuals with A(H7N9) or A(H1N1)pdm09 infections.^103^ No human infections with A(H5N2) or A(H5N8) viruses have been reported to date.

Although virologic surveillance is typically not designed to detect cases that are not severe such as influenza‐like illness, serologic studies can estimate the frequencies of less severe and mild infections. Two studies have looked for evidence of seroconversion to clade 2.3.4.4 A(H5) viruses in poultry farmers.^111^, ^112^ In a study involving 523 farmers exposed to poultry during the 2016‐2017 ROK A(H5N6) outbreaks, no evidence for infection was found when using a microneutralization assay to detect seropositivity.^111^ In another study, 61 of 760 sera from poultry farmers in the Russian Federation had hemagglutination inhibition titers greater than 20 against an A(H5N8) virus.^112^ In terms of preexisiting antibodies to A(H5) viruses in the general population, Freidl et al. were unable to detect reactivity against A(H5N1) antigens both before and after the A(H1N1)pdm09 pandemic in 6896 blood samples collected from 11 countries in Asia, Europe, and North America as tested with an HA protein microarray.^113^ Zhao et al. also showed that no neutralizing antibody against the A(H5N1) virus, A/Vietnam/1194/2004, was detected among 35 healthy volunteers in China.^114^ These data support the premise that there is a lack of immunity in the general population, which constitutes a significant risk, should the clade 2.3.4.4 A(H5) virus gain efficient human‐to‐human transmissibility.

The 22 A(H5N6) viruses from human cases for which genetic sequence data (GSD) are available in the EpiFlu database of GISAID were all classified as clade 2.3.4.4 A(H5) viruses. Subgroups within clade 2.3.4.4 to which the human viruses belong have changed over time: a virus collected in February 2014 belonged to the A/Sichuan/26221/2014‐like group, 20 viruses collected between April 2014 and November 2017 belonged to the A/Hubei/29578/2016 group, and a virus in the A/Fujian‐Sanyuan/21099/2017 group was detected in 2017 (Table 1). GSD of the most recent viruses are not yet available. With the exception of the 2014 virus, of available data so far, all human viruses had a NA stalk deletion at amino acid positions 58‐68, which is known to be an adaptation to terrestrial poultry and has been associated with enhanced virulence in mice presumably by altering the HA‐NA balance of the virus. Although all human infections were with A(H5N6) viruses, a number of different genotypes were involved containing a variety of internal genes originating from A(H5N1) and A(H9N2) viruses circulating in poultry, as well as A(H3) viruses circulating in ducks.^98^, ^102^, ^104^, ^115^ Multiple amino acid substitutions associated with mammalian adaptation were found in viral proteins, particularly in internal proteins. Amino acid substitutions that confer oseltamivir resistance (H274Y and N294S by N2 numbering) were not found in the human A(H5N6) virus isolates, consistent with their low frequency in avian origin viruses. Some strains of A(H5N6) from human cases did, however, have the M2 S31N mutation associated with adamantine resistance.

**Table 1:** Genomic characteristics of 22 human clade 2.3.4.4 A(H5N6) viruses

Year of isolation			Virus strains^a^^,^ b						
			*Sichuan/26221*	*Changsha/1, Gz/39715, Gd/99710*	*Yunnan/0127, Yunnan/DQ001, Yunnan/DQ002, Shenzhen/1/15, Shenzhen/TH001, Yunnan/14563, Yunnan/14564, Gd/SZ872, Gd/ZQ874*	*Shenzhen/TH002, Shenzhen/TH003, Shenzhen/1/16*	*Anhui/33162, Anhui/33163, Hubei/29578*	*Hunan/55555, Guangxi/55726*	Fujian‐Sanyuan/21099
			2014		2015	2016			2017
Gene	Phenotype	Amino acid position	Amino acid substitution						
HA^c^	Glycosylation site at 158	N158D	N	N	N	N	N	N	N
		T160A	A	A	A	A	S (Anhui/33162, Anhui/33163), A (Hubei/29578)	A	A
	Receptor binding specificity^d^	N186K	N	N	N	N	N	N	N
		N193K	N	N	N	N	N	D	N
		Q196R	K	K	K	K	K	K	K
		N224	N	N	N	N	N	N	N
		Q226L	Q	Q	Q	Q	Q	Q	Q
		G228S	G	G	G	G	G	G	G
		S227N	R	R	S (Shenzhen1/15, Shenzhen/TH001, Gd/SZ872), R (the others)	S	S	R	R
		S227R							
		T318I	T	T	T	T	T	T	T
	Cleavage site	335‐348	RERRRKR	RERRRKR	RERRRKR	RERRRKR	RERRRKR	RERRRKR	REKRRKR
NA^e^	Oseltamivir resistance	H274Y	H	H	H	H	H	H	H
		N294S	N	N	N	N	N	N	N
	Stalk deletion		No	58–68 deletion					
PB2	Increased pathogenicity in mice	E627K	E	E (Changsha/1), K (Gz/39715, Gd/99710)	E (Shenzhen/1/15, Shenzhen/TH001, Gd/SZ872, Gd/ZQ874), K (the others)	K	E (Hubei/29578), K (Anhui/33162, Anhui/33163)	E	E
		D701N	N	D	D	D	D	D	D
		Q591K	Q	Q	Q	Q	Q	Q	Q
		T/I271A	T	T	T	T	T	T	T
		558 V	E	E	E	E	E	E	E
PB1	Increased replication in mammalian cells	L473V	V	V	V	V	V	V	V
		L598P	L	L	L	L	L	L	L
PB1‐F2	Increased pathogenicity in mice		57	11	11 (Gd/ZQ874), 90 (the others)	90 (Shenzhen/TH002, Shenzhen/1/16), 76 (Shenzhen/TH003)	34 (Hubei/29578), 90 (Anhui/33162, Anhui/33163)	90	90
PA	Increased replication in mice	A36T	A	A	A	A	A	A	A
M1	Increased pathogenicity in mice	N30D	N	N	N	N	N	N	N
		T215A	A	A	A	A	A	A	A
M2	Adamantane resistance	S31N	S	S	S (Gd/ZQ874), N (the others)	N	N	S	S
NS	Increased pathogenicity in mice	P42S	S	S	S	S	S	S	S
	Deletion of aa 80‐84		Yes	Yes	Yes (Gd/ZQ874), No (the others)	No	No	Yes	Yes
	Increased pathogenicity in mice	D92E	E	E	E (Gd/ZQ874), D (the others)	D	D	E	D
	Presence of PDZ domain		Yes	Yes	Yes (Gd/ZQ874), No (the others)	No	No	Yes	Yes

a Font styles represent genetic groups to which the viruses tested belong; italic underline, A/Sichuan/26221/2014 group; italic, A/Hubei/29578/2016 group; underline, A/Fujian‐Sanyuan/21099/2017 group.

b Sichuan/26221, A/Sichuan/26221/2014; Gd/99710, A/Guangdong/99710/2014; Gz/39715, A/Guangzhou/39715/2014; Changsha/1, A/Changsha/1/2014; Yunnan/0127, A/Yunnan/0127/2015; Yunnan/DQ001, A/Yunnan/DQ001/2015; Yunnan/DQ002, A/Yunnan/DQ002/2015; Shenzhen/1/15, A/Shenzhen/1/2015; ShenZhen/TH001, A/ShenZhen/TH001/2015; Yunnan/14563, A/Yunnan/14563/2015; Yunnan/1456, A/Yunnan/14564/2015; Gd/SZ872, A/_Guangdong_/SZ872/2015; Gd/ZQ874, A/_Guangdong_/ZQ874/2015; Shenzhen/1/16, A/Shenzhen/1/2016; Shenzhen/TH003, A/Shenzhen/TH003/2016; Shenzhen/TH002, A/Shenzhen/TH002/2016; Hunan/55555, A/Hunan/55555/2016; Guangxi/55726, A/Guangxi/55726/2016; Hubei/29578, A/Hubei/29578/2016; Anhui/33162, A/Anhui/33162/2016; Anhui/33163, A/Anhui/33163/2016; Fujian‐Sanyuan/21099, A/Fujian‐Sanyuan/21099/2017.

c Amino acid positions of HA protein are designated by H3 numbering.

d Amino acid substitution Q226L/G228S and N224K/Q226L in HA are responsible to increase the ability to bind to human‐type receptors in combination.

e Amino acid positions of NA protein are designated by N2 numbering.

## 5 RECEPTOR BINDING PROPERTIES OF CLADE 2.3.4.4 A(H5) VIRUSES

The specificity of the viral HA for the host cell receptor molecule regulates virus entry into cells. Human influenza A viruses preferentially bind to receptors with sialic acid linked to galactose by an α2,6 linkage (Sia‐α2,6Gal), which is abundantly displayed in the upper respiratory tract of humans.^116^ In contrast, most avian influenza A viruses have a binding preference for receptors with Sia‐α2,3Gal, which is sparse in the upper respiratory tract of humans, but abundant in the intestinal mucosa of birds.^116^ The difference in receptor binding preference is considered to be one of the main reasons why avian viruses rarely infect and transmit poorly in humans and human influenza viruses do not replicate well in birds.

Among 1994 clade 2.3.4.4 A(H5) viruses isolated between January 2013 and October 2018 with GSD available in the EpiFlu database of GISAID, 1988 (99.7%) had an HA‐160A (H3 numbering) amino acid residue and 1295 (64.8%) had HA‐227R amino acid residue (Supplementary Table 2). The HA‐160A substitution results in lack of a glycosylation motif in combination with residues 158‐160 of HA1, which facilitate airborne transmission in ferrets (HA‐N158D by Imai et al. and HA‐T160A by Herfst et al. in H3 numbering).^117^, ^118^, ^119^ Amino acid residues at positions 222 and 227 play important roles in binding sialyl Lewis X (3′SLeX), which is abundant on the epithelial cells of the chicken trachea.^120^ Overall, there was no notable difference in the GSD of the HA receptor binding site among A(H5N2), A(H5N6), and A(H5N8) viruses despite only A(H5N6) viruses being found in human infections (Supplementary Table 2). Among the studies that examined receptor binding specificity of clade 2.3.4.4 A(H5) viruses, 13 isolates including two A(H5N2), seven A(H5N6), and four A(H5N8) viruses had receptor binding specificity for both Sia‐α2,6Gal and Sia‐α2,3Gal (Table 2). In general, the viruses that exhibited affinity for human‐type receptors also maintained a high affinity for avian‐type receptors. It is thought that a human transmissible virus could only have low affinity for the avian‐type receptor. Most of the 13 viruses with dual receptor specificity had HA amino acids 128P, 137A, and 160A, but not all viruses possessing these amino acids had dual‐receptor specificity (Table 2). Additional amino acid substitutions needed to cross the species barrier likely vary with the makeup of the HA gene. Biophysical assays such as glycan arrays, solid‐phase binding assays, and HA assays using sialidase‐treated red blood cells have been mainstream methods to analyze the receptor binding specificity of influenza viruses. Virus tropism in ex vivo cultures of human bronchus has also been suggested to be an alternative experimental model to assess receptor binding of animal viruses to the human respiratory tract.^121^ Only a few glycans present in glycan arrays are present on the human respiratory tract.^122^ Similarly, A/environment/Korea/W541/2016 (H5N6), although not possessing known molecular markers associated with mammalian adaptation (namely PB2 627K, 271A, 590S, 591R, 147T, 339T, or 588T), replicated well in human NHBE cells and ex‐vivo lung tissues.^84^ Moreover, A/Guangzhou/39715/2014 A(H5N6), which was shown to predominantly bind to Sia‐α2,3 Gal and possessed PB2 627K, grew comparably to an A(H1N1)pdm09 virus in ex vivo human bronchus and lung culture^123^ (Table 2). A/Fujian‐Sanyuan/21099/2017‐like A(H5N8) viruses, in contrast, replicated poorly in ex vivo cultures of human lung explants.^97^

**Table 2:** Receptor binding specificities of the clade 2.3.4.4 A(H5) virus characterized by biophysical assays

Reference	Strain name^c^	Subtype	Method	Results^b^		Avian	Amino acid residue in H3 numbering											
							101	128	137	158	160	193	196	224	226	227	228	318
							Amino acid substitution^a^											
							D	S	S	N	T	N	Q	N	Q	S	G	T
				SA‐a2,6Gal	SA‐a2,3Gal	Human	N	P	A	D	A	K	R/H	K	L	N/R	S/A	I
Li et al.^124^	*A/duck/Eastern China/1111/2011*	H5N2	Solid phase binding assay	○	○		N	P	A		A		K			R		
	*A/goose/Eastern China/1112/2011*			○	○		N	P	A		A		K			R		
Kaplan et al.^88^	**A/Northern pintail/Washington/40964/2014**			×	○		T	P	A		A		K					
Yang et al.^136^			Glycanarray	×	○		T	P	A		A		K					
Kwon et al.^84^	*A/environment/Korea/W541/2016*	H5N6	Solid phase binding assay	×	○		N	P	A		A		K			Q		
Sun et al.^94^	*A/duck/Anhui/S4/2016*			○	○^d^		N	P	A		A		K			R		
	*A/goose/Yangzhou/YZ587/2016*			○	○^d^		N	P	A		A		K			R		
	*A/Chicken/Guangdong/GD1602/2016*			○	○^d^		N	P	A		A		K					
	*A/Chicken/Xuzhou/XZ6/2016*			○	○^d^		N	P	A		A		K			R		
Liu et al.^137^	*A/chicken/Anhui/MZ33/2016*			○	○		N	P	A		A		K					
	*A/ chicken/Anhui/MZ34/2016*			○	○		N	P	A		A		K					
	*A/chicken/Henan/YB0597/2016*			○	○		N	P	A		A		K					
Herfst et al.^86^	*A/Guangzhou/39715/2014*		Sialidase‐treated HA assay	×	○		N	P	A				K					
Hui et al.^123^				×	○													
Yang et al.^136^	*A/Sichuan/26221/2014*		Glycanarray	×	○		N	T	A		A		K			R		
Li et al.^124^	**A/duck/Jiangsu/k1203/2010**	H5N8	Solid phase binding assay	○	○		S	P	A		A		K			R		
Kwon et al.^84^	**A/Common teal/Korea/W555/2017**			×	○		S	P	A		A		K			Q		
Kaplan et al.^88^	**A/mallard/Korea/W452/2014**			×	○		T	P	A		A		K			R		
	**A/chicken/Kumamoto/1–7/2014**			×	○		T	P	A		A		K			R		
	**A/duck/England/36254/2014**			×	○		T	P	A		A		K			R		
	**A/gyrfalcon/Washington/41088–6/2014**			×	○		T	P	A		A		K					
Yang et al.^136^			Glycanarray	×	○													
Li et al.^124^	**A/duck/Shandong/Q1/2013**		Solid phase binding assay	○	○		S	P	A		A		K			R		
Fan et al.^87^	**A/mallard duck/Shanghai/SH‐9/2013**			×	○		S	L	A		A		K			R		
Wang et al.^93^	**A/goose/Eastern China/CZ/2013**		Sialidase‐treated HA assay	○	○		S	L	A		A		K			R		
	**A/duck/Eastern China/JY/2014**			○	○		S	P	A		A		K			R		

a Dots represent amino acid residues that are conserved among avian species.

b ○ denotes the virus tested bound to SA‐a2,3Gal or SA‐a2,6Gal; × denotes the virus tested did not bind to SA‐a2,3Gal or SA‐a2,6Gal.

c Font styles represent genetic groups to which the viruses tested belong; italic underline, A/Sichuan/26221/2014 group; bold, A/gyrfalcon/Washington/41088‐6/2014 group; italic, A/Hubei/29578/2016 group; underline, A/Fujian‐Sanyuan/21099/2017 group.

d The virus tested showed the dual specificity both to SA‐a2,3Gal and SA‐a2,6Gal with stronger affinity to SA‐a2,3Gal than SA‐a2,6Gal.

## 6 ASSESSMENT OF THE TRANSMISSIBILITY OF THE CLADE 2.3.4.4 A(H5) VIRUSES IN ANIMAL MODELS

Several studies have been conducted to assess the transmissibility of the clade 2.3.4.4 A(H5) viruses. These included assessing direct contact and respiratory droplet transmission using multiple animal models, namely ferrets, pigs, guinea pigs, and dogs^84^, ^86^, ^88^, ^89^, ^90^, ^91^, ^92^, ^95^, ^96^, ^97^, ^124^, ^125^ (Table 3). Five clade 2.3.4.4 A(H5) viruses, including one of which preferentially bound to Sia‐α2,3 Gal^84^ and one of which showed dual‐receptor specificity,^124^ were transmitted via direct contact in guinea pig or ferret models^84^, ^95^, ^124^, ^125^ (Table 3). An A(H5N6) virus A/environment/Korea/W541/2016, which grew well in human cells despite having strong affinity to avian‐type receptors, transmitted to two of three ferrets co‐housed with infected animals.^84^ The high proliferation competency of this virus strain in human NHBE cells and ex vivo lung tissues might have facilitated its transmission via direct contact. Herft et al. demonstrated that the HA of A/Guangzhou/39715/2014 A(H5N6) showed less acid stability than an A/Indonesia/5/2005 A(H5N1) virus adapted for airborne‐transmission between ferrets and an H3N2 seasonal influenza virus, A/Netherlands/213/2003.^86^ Correspondingly, A/Guangzhou/39715/2014 A(H5N6), which exclusively bound to Sia‐α2,3 Gal, did not transmit among ferrets via respiratory droplets. The individual infected with this A(H5N6) virus had underlying disease and exposure to infected poultry which might have promoted virus replication competency in human cells and infection. Airborne or respiratory droplet transmission of clade 2.3.4.4 A(H5N2), A(H5N6), and A(H5N8) viruses has not been demonstrated in any animal model examined, which is consistent with the epidemiology of the virus in humans (showing no evidence of human‐to‐human spread) (Table 3).

**Table 3:** Transmission of clade 2.3.4.4 A(H5) viruses in mammalian animal models

Reference	Strain name^c^		Results of receptor binding property^a^		Result of transmission studies^b^		
		Subtype	SA‐a2,6Gal	SA‐a2,3Gal	Animal model assessed	Direct contact	Respiratory droplet
Li et al.^124^	***A/goose/Eastern China/1112/2011***	H5N2	○	○	Guinea Pig	○	N/A
Pulit‐Penaloza et al.^89^	A/northern pintail/Washington/40964/2014		×	○	Ferret	×	N/A
Kaplan et al.^88^			×	○	Ferret	×	N/A
			×	○	Pig	×	N/A
	A/turkey/Minnesota/7172‐1/2015		N/A	N/A	Pig	×	N/A
Sun et al.^125^	**A/duck/Eastern China/S0711/2014**	H5N6	N/A	N/A	Ferret	○	×
Kwon et al.^84^	**A/Common teal/Korea/W555/2017**		×	○	Ferret	×	×
Sun et al.^125^	*A/goose/Eastern China/S0513/2013*		N/A	N/A	Ferret	○	×
Noh et al.^90^	*A/Mandarin duck/Korea/K16‐187‐3/2016*		N/A	N/A	Ferret	×	×
Kwon et al.^84^	*A/environment/Korea/W541/2016*		×	○	Ferret	○	×
Herfst et al.^86^	*A/Guangzhou/39715/2014*		×	○	Ferret	N/A	×
Zhao et al.^95^	*A/duck/Hubei/XY‐01/2016*		N/A	N/A	Guinea Pig	×	×
	*A/chicken/Hubei/XY‐165/2016*		N/A	N/A	Guinea Pig	○	×
	*A/chicken/Hubei/XY‐918/2016*		N/A	N/A	Guinea Pig	×	×
Lyoo et al.^96^	*A/chicken/VN/LangSon/P140450/2014*		N/A	N/A	Dogs	×	
Pulit‐Penaloza et al.^89^	**A/Gyrfalcon/Washington/41088‐6/2014**	H5N8	×	○	Ferret	×	N/A
			×	○	Pig	×	N/A
Kaplan et al.^88^	**A/chicken/Kumamoto/1‐7/2014**		×	○	Ferret	×	N/A
	**A/duck/England/36254/2014**		×	○	Ferret	×	N/A
Richard et al.^91^	**A/Chicken/Netherlands/EMC‐3/2014**		N/A	N/A	Ferret	N/A	×
Grund et al.^97^	**A/tufted duck/Germany/AR8444‐L01987/2016**		N/A	N/A	Ferret	×	N/A
Kaplan et al.^88^	*A/mallard/Korea/W452/2014*		×	○	Ferret	×	N/A
Kim et al.^92^			×	○	Ferret	N/A	×

a ○ denotes the virus tested bound to SA‐a2,3Gal or SA‐a2,6Gal; × denotes the virus tested did not bind to SA‐a2,3Gal or SA‐a2,6Gal; N/A, not assessed.

b ○ denotes that transmission by direct contact or respiratory droplets; × denotes that transmission by direct contact or respiratory droplet was not observed; N/A, not assessed.

c Font styles represent genetic groups to which the viruses tested belong; italic underline, A/Sichuan/26221/2014 group; bold, A/gyrfalcon/Washington/41088‐6/2014 group; italic, A/Hubei/29578/2016 group; underline, A/Fujian‐Sanyuan/21099/2017 group.

Receptor binding affinity is a prerequisite, but insufficient alone to promote airborne transmission of A(H5) avian viruses. Several studies have shown that compensatory mutations in HA are required to counteract the HA instability caused by human‐type receptor‐binding mutations.^117^, ^118^, ^126^ Additional mutations are also involved to increase viral proliferation and transcription.^117^, ^119^ Identified compensatory mutations to enhance thermostability and facilitate membrane fusion at a lower pH are located in both the globular head and stalk regions of the HA.^119^, ^127^ Chen et al. suggested that optimization of HA, NA, and internal genes is a requirement for efficient transmission.^128^ They demonstrated that an A(H5N1) reassortant virus with Sia‐α2,6 Gal preferential binding (amino acid substitutions Q196R, Q226L, G228S) coupled with the NA of a human seasonal A(H3N2) virus was transmitted via respiratory droplets among ferrets, whereas the same virus combined with the NA of the avian A(H5N1) virus was not transmitted.^128^ Furthermore, internal genes also contribute undetermined functions that lead to efficient transmission. Zhang et al. demonstrated that the NS gene of the A(H1N1)pdm09 virus enabled a reassorted A(H5N1) virus to efficiently transmit among guinea pigs via respiratory droplets but the avian NS gene did not.^126^ A scenario in which a clade 2.3.4.4 A(H5) virus reassorts with a human seasonal influenza virus may facilitate transmission among mammals, although further adaptations would likely be needed for optimal spread. Taken all together, and reassuringly, clade 2.3.4.4 A(H5N2), A(H5N6), and A(H5N8) viruses have so far shown limited ability to infect and transmit efficiently in mammalian species.

## 7 DISCUSSION

The experimental data generated to date has not detected differences in receptor binding specificity and transmission capability among mammals between clade 2.3.4.4 A(H5N2), A(H5N6), and A(H5N8) viruses despite that only the A(H5N6) subtype of the clade 2.3.4.4 A(H5) viruses among three has been found in humans. Dual‐receptor binding specificity, viruses that show equal binding in vitro to both human and avian receptor analogues, has been observed in viruses of all three subtypes, and some of the viruses were transmitted via direct contact among ferrets or guinea pigs. However, no studies have identified receptor binding profiles showing a preference for binding to human receptor analogues, or animal model transmission patterns, showing spread via the aerosol route, consistent with a virus adapted to transmit in humans. What is less clear is precisely which molecular changes would lead to such adaptation.

A/Hubei/29578/2016‐group clade 2.3.4.4 A(H5) viruses, which are primarily A(H5N6) viruses, have been confined to Asia. In contrast, A/gyrfalcon/Washington/41088‐6/2014‐group A(H5N2) and A(H5N8) viruses and A/Fujian‐Sanyuan/21099/2017‐group A(H5N8) viruses spread from Asia to North America, Europe, the Middle East, and the African continent and gave rise to numerous outbreaks among poultry and wild birds following reassortment with viruses from local avian species. Despite significant exposure to A(H5N2), A(H5N6) or A(H5N8) infected poultry, so far only the A(H5N6) subtype viruses have caused human infection. What then differentiates the zoonotic potential of the A(H5N6) viruses from that of the other two subtypes?

Several possible reasons can be considered here. First, biosecurity systems vary across countries. A/Fujian‐Sanyuan/21099/2017‐group A(H5N8) and A/gyrfalcon/Washington/41088‐6/2014‐group A(H5N2) and A(H5N8) viruses were detected in poultry in multiple places in the United States and in Europe, resulting in severe impacts on the poultry industries. The majority of affected countries executed a systematic stamping‐out strategy.^129^ If outbreaks of the same magnitude as the A(H5N2) outbreaks in the United States during 2014‐2015 had happened in regions where biosecurity and precautionary strategies were less stringent, the risks of human infection might have been higher. Perhaps more importantly, live poultry markets, which are a significant source of human exposure in Asia, are rare in the United States and Europe, limiting highly contaminated environments inhabited by birds with humans in close contact. Second, more controversially, inadequate or improper vaccination in poultry can also complicate eradication of HPAI. Between 2002 and 2010, 15 countries implemented vaccination in poultry against HPAI A(H5N1) or A(H7) avian influenza viruses as food security and animal health measures within a long‐term control program.^130^ In one example, when vaccines were antigenically similar with the targeted A(H5N1) viruses and were properly applied with production of a protective immune response in ≥60% of the poultry population, a reduction in virus infection and transmission was achieved and outbreaks declined.^131^ When a protective immune response was produced in <60% of the poultry population or the vaccine was antigenically less similar to the field viruses, the A(H5N1) viruses were able to breakthrough vaccinated population and result in additional outbreaks.^131^ In the latter scenario, A(H5N1) infected birds with no disease signs had been sent to market, resulting in infection and propagation of the virus within the market environment raising the risk of human infection. In 2017, after the emergence of the HPAI A(H7N9) variants, China added an H7 antigen to the existing monovalent H5 vaccines used in poultry. The bivalent H5/H7 vaccine was introduced in Guangdong and Guangxi provinces in July 2017, followed by introduction into other regions by the winter of 2017‐2018. The number of reported human A(H7N9) cases were reduced by 92% after the enhanced poultry vaccination campaign, and only one human H7N9 infection has been reported to WHO since 2019 to the present.^132^ However, some countries lack the financial and human resources for a comprehensive stamping‐out program.

Last, although different exposures to infected poultry may have contributed to human infections with the specific subtype A(H5N6), it is also possible that biological features of A(H5N6) viruses also contributed to occurrence of human infections. In a study conducted by Chen et al., an A(H5N1) reassortant virus with several amino acid substitutions in HA and the NA gene of a human seasonal A(H3N2) virus (A/Brisbane/10/2007) was transmitted via respiratory droplets between ferrets, but an A(H5N1) reassortant virus with the same HA gene and the NA gene of a human seasonal A(H1N1) virus (A/Brisbane/59/2007) was not transmitted.^128^ Additional studies, including gain‐of‐function (GOF) research, are instrumental to better elucidate the potential mechanism that allows some viruses to cross species barriers.

The importance of continued monitoring of the ecology and ongoing evolution of potentially zoonotic avian influenza viruses should not be underestimated. Some of the fundamental and important activities such as surveillance programs in diverse animal reservoirs, including wildlife, are not always a high priority and properly funded. Several studies on poultry outbreaks caused by clade 2.3.4.4 A(H5) viruses in the United States and the Netherlands suggested that the viruses were introduced from wild birds rather than farm‐to‐farm transmissions.^29^, ^133^ In a review, Morin et al. has warned that accelerated warming of the Arctic by climate change has the potential to affect migratory patterns, the timing of biological events, and habitats of migratory birds, resulting in the potential to impact virus transmission dynamics among avian species.^134^ The surveillance activities should incorporate a component of how environmental changes may affect influenza virus hosts and the distribution and genomic constellations of influenza A viruses.

## 8 CONCLUSION

Because their emergence, the clade 2.3.4.4 HPAI A(H5) viruses have evolved through point mutations and reassortment with circulating local viruses following global expansion via distinct pathways. So far, these viruses have caused only sporadic human infections and are unable to transmit efficiently among humans. Studies have shown that some clade 2.3.4.4 A(H5) viruses have dual‐receptor specificity and can transmit between ferrets in direct contact. Furthermore, some A(H5N6) viruses isolated from humans have molecular signatures related to mammalian adaptation. It is uncertain what other changes are necessary for these viruses to become transmissible among humans. Their widespread distribution, ongoing evolution, and periodic infection of mammalian hosts increase the chances that efficient transmissibility is possible to be acquired. This calls for surveillance of influenza viruses in domestic and wild birds to be enhanced to allow for timely development and updating of veterinary and public health countermeasures and to reduce the threats of zoonotic and pandemic influenza.

## 9 GENOMIC ANALYSIS

HA genetic sequence data (GSD) of HPAI viruses isolated from animals, including mammals and avian species, that possessed multibasic amino acids at the HA cleavage site between 2013 and February 2019 and available in the EpiFlu database of Global Initiative on Sharing All Influenza Data (GISAID) were analyzed. Between 2013 and February 2019, 2553 A(H5) HPAI viruses were available. The open reading frame of the HA genes of A(H5) viruses was used for phylogenetic analysis. Multiple sequence alignment of H5 viruses was performed using BioEdit 7.2. A maximum‐likelihood tree was constructed for MEGA 7 with 1000 replicates. The 242 virus GSDs were used as the reference for the nomenclature of A(H5) HA systematized by World Health Organization/World Organization for Animal Health/Food and Agriculture Organization (WHO/OIE/FAO) H5 Evolution Working Group.^135^ The phylogenetic tree is available upon request. Among the 2553 H5 viruses, 1994 H5 viruses, which belonged to clade 2.3.4.4, were used further analysis.

## CONFLICT OF INTERESTS

JSMP has received research funding from Crucell NV and is ad‐hoc consultant for GlaxoSmithKline and Sanofi. YK has received speaker's honoraria from Toyama Chemical and Astellas; grant support from Chugai, Daiichi Sankyo, Toyama Chemicals, Tauns, Tsumura, and Denka Seiken and is a co‐founder of FluGen.

## AUTHORS' CONTRIBUTIONS

RY drafted the manuscript and constructed the tables and figures with contributions from MDS, CTD, DES, DW, FYKW, JWM, JSMP, RJW, RAMF, YK, and WZ in reviewing results, reviewing the manuscript, and providing suggestions. RY, MDS, and WZ managed the project.

## Supporting information


**Supplementary Table 1** A(H5N6) viruses with the A/Fujian‐Sanyuan/21099/2017‐like HA gene for which sequences are available in the EpiFlu database of GISAID

**Supplementary Table 2** Amino acid substitutions related to receptor binding specificity among clade 2.3.4.4 A(H5) avian viruses available from GISAID as of February 2019
