# Supplementary material for: Pandemic potential of highly pathogenic avian influenza clade 2.3.4.4 A(H5) viruses
Source: Rev Med Virol. 2020 Mar 5;30(3):e2099. doi: 10.1002/rmv.2099 (PMC9285678; doi:10.1002/rmv.2099)
Supplement: Supplementary file 1 — Supplementary Table 1 A(H5N6) viruses with the A/Fujian‐Sanyuan/21099/2017‐like HA gene for which sequences are available in the EpiFlu database of GISAID [file RMV-30-0-s002.docx]

**Supplemental Table 1**

**A(H5N6) viruses with the A/Fujian-Sanyuan/21099/2017-like HA gene for which sequences are available in the EpiFlu database of GISAID**

| **Countries isolated** | **Strain name** |
| --- | --- |
| Greece | A/chicken/Greece/39 2017/2017 |
| Japan | A/tufted duck/Shimane/3211TY001/2017 |
|  | A/jungle crow/Hyogo/2803E011/2018 |
|  | A/jungle crow/Hyogo/2803E022/2018 |
|  | A/jungle crow/Hyogo/2803A002/2018 |
|  | A/mute swan/Shimane/3211A001/2017 |
| Germany | A/common pochard/Germany-BY/AR09-18-L02421/2017 |
| Republic of Georgia | A/Armenian Gull/Republic of Georgia/4/2017 |
|  | A/Mallard/Republic of Georgia/1/2018 |
| Denmark | A/white-tailed eagle/Denmark/3073-1w/2018 |
